# Supplementary material for: Smooth Interpolating Curves with Local Control and Monotone Alternating Curvature
Source: Comput Graph Forum. 2022 Oct 6;41(5):25–38. doi: 10.1111/cgf.14600 (PMC9827861; doi:10.1111/cgf.14600)
Supplement: Supplementary file 1 — Supplement Material [file CGF-41-25-s001.zip › Local-Smooth-Interpolating-MonoCurvature/extern/clothoids/docs/api-matlab/root.html]

MATLAB API — Clothoids v2.0.9

### Navigation

- index
- toc
- next
- previous
- Clothoids »
- MATLAB API

# MATLAB API¶

## Class Hierarchy¶

- - Class Biarc
  - Class BiarcList
  - Class CircleArc
  - Class ClothoidCurve
  - Class ClothoidList
  - Class ClothoidSplineG2
  - Class CurveBase
  - Class LineSegment
  - Class PolyLine
  - Class Triangle2D

## File Hierarchy¶

- - File Biarc.m
  - File BiarcList.m
  - File CircleArc.m
  - File ClothoidCurve.m
  - File ClothoidList.m
  - File ClothoidSplineG2.m
  - File CompileOctave.m
  - File CurveBase.m
  - File LineSegment.m
  - File PolyLine.m
  - File Triangle2D.m
  - File TriTriOverlap.m

## Full API¶

### Classes and Structs¶

- Class Biarc
  - Inheritance Relationships
    - Base Type
  - Class Documentation

- Class BiarcList
  - Inheritance Relationships
    - Base Type
  - Class Documentation

- Class CircleArc
  - Inheritance Relationships
    - Base Type
  - Class Documentation

- Class ClothoidCurve
  - Inheritance Relationships
    - Base Type
  - Class Documentation

- Class ClothoidList
  - Inheritance Relationships
    - Base Type
  - Class Documentation

- Class ClothoidSplineG2
  - Inheritance Relationships
    - Base Type
  - Class Documentation

- Class CurveBase
  - Inheritance Relationships
    - Base Type
    - Derived Types
  - Class Documentation

- Class LineSegment
  - Inheritance Relationships
    - Base Type
  - Class Documentation

- Class PolyLine
  - Inheritance Relationships
    - Base Type
  - Class Documentation

- Class Triangle2D
  - Inheritance Relationships
    - Base Type
  - Class Documentation

### Functions¶

- Function TriTriOverlap
  - Function Documentation

### Quick search

### Table of Contents

- Matlab Interface Manual
- C++ API
- MATLAB API
  - Class Hierarchy
  - File Hierarchy
  - Full API

«
hide menu

menu
sidebar
»

### Navigation

- index
- toc
- next
- previous
- Clothoids »
- MATLAB API

© Copyright 2021, Enrico Bertolazzi and Marco Frego.
Created using Sphinx 4.2.0.
